# Triple Fluorescence staining to Evaluate Mechanism-based Apoptosis following Chemotherapeutic and Targeted Anti-cancer Drugs in Live Tumor Cells

**DOI:** 10.1038/s41598-018-31575-3

**Published:** 2018-09-04

**Authors:** Pradip De, Jennifer H. Carlson, Brian Leyland-Jones, Casey Williams, Nandini Dey

**Affiliations:** 1 Department of Molecular & Experimental Medicine, Avera Research Institute, Sioux Falls, SD USA; 2 Department of Internal Medicine, University of South Dakota SSOM, USD, Sioux Falls, SD USA

## Abstract

We present a protocol for live cancer cell-imaging by triple-fluorescent staining to test 3 crucial mechanisms of apoptosis; the enzymatic activity of executioner caspase3, caspase-dependent phosphatidylserine presentation on the cell surface and mitochondrial function. We standardized a protocol to co-stain live tumor cells with the NucView488-Casp3 substrate, CF594 AnnexinV, and MitoViewBlue. We validated this protocol following apoptosis induction with paclitaxel or in combination with BKM120. Fluorescent imaging of cells using simultaneous live/dead cell markers (CalceinAM green/EthD-1red) was used as internal control. We used quantitative confluence (Essen), AnnexinV-PE staining (Accuri C6), expression of cl-caspase3, Cl-PARP and mitochondrial potential (TMRE-A) as validation criteria in A2780 and OVK18 cells following drug treatment which decreased proliferation, & increased apoptotic signaling with mitochondrial depolarization. Treatment blocked cytoplasmic MitoViewBlue staining while increased both nuclear NucView488-Casp3 substrate and red membranous CF594 AnnexinV staining. Merged images showed 100% mutual exclusivity between MitoViewBlue and caspase3 or AnnexinV stains in control and treated cells as determined by overlap and colocalization coefficients. Caspase3 and AnnexinV staining in treated cells were both separate and overlapped (yellow fluorescence) indicating the sequence of apoptotic-events. The protocol will help in deciphering mechanistic involvement of different stages/features of apoptosis in tumor cell following anti-cancer drugs in real-time.

## Introduction

Apoptosis is a morphologically distinct form of cell death^1^. Inhibition of apoptosis is a trait commonly shared by tumor cells providing them with survival advantages and enabling the tumor to evolve to higher stages and metastatic states.

Apoptosis is a genomically regulated, and proteomically coordinated energy-dependent process that involves characteristic cytomorphological features and biochemical events which are tightly regulated in an ATP-dependent manner leading to a time-dependent sequence of events to activate the cysteine family of proteases called caspases.

The spectrum of cytomorphological features of apoptosis in a timeline includes; cell shrinkage, rounding, and pyknosis of nucleus due to chromatin condensation, loss of nuclear membrane integrity, plasma membrane blebbing followed by karyorrhexis and separation of cell fragments into apoptotic bodies called “budding”^2^. The biochemical events include; initiator and effector caspase activation, mitochondrial membrane-alterations, the release of cytochrome C from mitochondria, externalization of phosphatidylserine on the plasma membrane, poly (ADP-ribose) polymerase (PARP) cleavage and internucleosomal DNA fragmentation. The main pathways of apoptosis are the extrinsic, intrinsic and the perforin/granzyme pathway. Each requires specific triggering signals to initiate its own energy-dependent cascade (initiator caspase, 8, 9, 10) of molecular events which in turn will activate the executioner caspase-3 at the final step. Both extrinsic, and intrinsic pathways of apoptosis converge on the execution pathway initiated by the cleavage of caspase-3/7 and result in DNA fragmentation, degradation of cytoskeletal and nuclear proteins, crosslinking of proteins, the formation of apoptotic bodies finally followed by the flipping of phosphatidylserine on the outer surface of the plasma membrane for phagocytic cell recognition.

Like any other zero-error biological event in nature, apoptosis is well coordinated to the energy synthesis machinery of the cell, mitochondria, mitochondrial potential and cytochrome C release. As an energy-dependent process, a distinctive feature of apoptosis is the interference of normal mitochondrial function, and mitochondria-dependent intrinsic signaling pathways are recognized as a component of apoptosis^3^, hence targeting mitochondria in cancer cells has been considered as an attractive therapeutic strategy^4,5^. Compromised mitochondrial (trans)membrane potential (ΔΨm) and its collapse lead to the opening of mitochondrial permeability transition pores, and the subsequent release of cytochrome C in the cytosol, which in turn initiates penultimate downstream events in the apoptotic cascade of caspases. Caspases cleave proteins at aspartic acid residues and initiate a complex cascade of proteolytic events. Activation of caspases sets an irreversible and rate-limiting step for the cell to undergo apoptosis^2^. Caspase activation cascade amplifies the signal and enzymatically links the initiating stimuli (intracellular/extracellular) to the final demise of the cell and its physiological scavenging via specific cell surface marker (“eat-me”) signals^2^ to the wandering or residential macrophages, parenchymal cells, or neoplastic cells. The extent of availability of intracellular ATP and executioner caspases are two cardinal factors that determine and distinguish a focal apoptotic process from widespread uncontrolled and passive necrosis^6,7^. Apoptosis had been long recognized and accepted as one of the distinctive and important modes of “programmed” cell death in cancer cells, which involves the genetically determined elimination of cells^8^.

In the light of the importance of all the above mechanism-based signals of apoptosis and anti-tumor drugs’ capacity to induce apoptosis in tumor cells, here we present a protocol to study three crucial mechanisms of apoptosis by triple fluorescence in live cancer cells. The three crucial mechanisms of apoptosis are (1) enzyme activity of executioner caspase3, (2) caspase-dependent phosphatidylserine exposure on the cell surface and (3) functional mitochondria. We standardized a protocol for co-fluorescence staining of live tumor cells using NucView488 Casp3 substrate, CF594 AnnexinV, and MitoView Blue and tested the effect of paclitaxel alone and in combination with PI3K pathway targeted anti-cancer drug, BKM120 in ovarian cancer cell models. The effect of paclitaxel alone and in combination with BKM120 in ovarian cancer cells was validated separately by real-time proliferation, apoptosis, mitochondrial potential, expression of apoptotic markers by WB, and staining of live-dead cells. Staining that identifies a live cell with live mitochondrial stain and the staining that identifies an apoptotic cell (with active caspase3 stains and annexin V stains) are mutually exclusive. Based on the above fact, we have also semi-quantified the colocalization characteristics of the triple fluorescence cells following drug treatments. Here we present a mechanistic application of the co-fluorescence (three) staining to evaluate apoptosis in live tumor cells following treatment with chemotherapeutic and targeted anti-cancer drug.

## Materials and Method

### Cell Culture, Reagents, and Antibodies

Ovarian cancer cell line OVK18 was obtained from RIKEN BRC Cell Bank, Japan. A2780 was procured from the American Type Culture Collection (ATCC, Manassas, VA). Cell lines were cultured according to standard protocols. Antibodies against cleaved-caspase3, cleaved PARP and BIM were procured from Cell Signaling Technology, Danvers, MA. For fluorescence staining, MitoViewBlue, NucView488, and CF594 AnnexinV were procured from Biotium, Inc. Fremont, CA, USA.

### Triple Fluorescence Staining of Live Cells

For the standardization of MitoViewBlue staining, a stock solution of 200 µM was made in DMSO and stored in a desiccator at −20 °C in single-use aliquots in a light protected storage conditions. The final concentration used was 50 nM in the culture media and 1X binding buffer for the standardization purpose. The 1X binding buffer was made in molecular grade water diluting the 5X binding buffer which was procured as a component (component 99966) of NucView® 488 and CF®594 Annexin V Dual Apoptosis Assay Kit (Catalog Number: 30067; Biotium). The live cells were incubated for 15–30 minutes in the dark and were photographed within 10 minutes. For the standardization of NucView488 caspase3 substrate staining, a stock solution of 0.2 mM was made in DMSO. Working concentration of 5 µM (1–10 µM) was used in the culture media and 1X binding buffer for the standardization purpose. For the standardization, 10 µM of caspase3 inhibitor Ac-DEVD-CHO was used. For the standardization of CF594 AnnexinV staining, a stock solution in TE buffer (as supplied by the manufacturer) was used. For triple fluorescence, a “staining mixture” was made in the dark with 1000 ml of 1X Binding buffer containing a range of 25–50 nM of MitoViewBlue and 5 µl of the manufacturer supplied NucView488 caspase3 substrate and 5 µl of the manufacturer supplied CF594 AnnexinV. Live cells were washed with sterile PBS (Phosphate Buffer Saline) twice and then rinsed with 1X Binding Buffer. Cells were then incubated with the “staining mixture” in the dark for 15–30 minutes. At the end of the incubation period, pictures were taken (Olympus DP72 digital camera) within 10 minutes (no wash). The cells were never fixed and stained for any counterstains.

### Real-time Confluency Assay and Apoptosis

The real-time proliferation of cells was performed using time-lapse phase-contrast imaging (IncuCyte; ESSEN BioScience). Proliferation was assessed using real-time microscopic images taken at 10x every 6 hours in an Essen IncyCyte Zoom. Cells were plated at a low confluence on day one, then treated with the indicated compounds on day two and imaged for up to 100 hours post-treatment. IncuCyte Zoom software was used to draw a mask to measure cell confluence. Cells were plated in quadruplicate. Using live cells, we generated long-term growth and growth inhibition curves and monitored morphology. The time course of the percentage of the confluence of non-treated and treated ovarian cancer cells (mean vs. time) was represented by four days (time courses: 24 hours, 48 hours, 72 hours and 96 hours)^9^. Cell cycle was tested as an internal control (data not shown). At the end of the treatment period, cells were trypsinized, fixed, and permeabilized in cold 70% ethanol and then washed with RPMI (phenol red-free with 1% FBS) and stained with propidium iodide/plus RNaseA (BD Biosciences, San Jose CA) before analysis by flow cytometry (Accuri C6). Apoptosis was measured using BD AnnexinV-PE and 7aaD apoptosis kit. Cells were treated in triplicate with the indicated compounds and analyzed at 48 and 72 hours post-treatment. Cells were trypsin-released, rinsed in FACS buffer, stained with AnnexinV-PE and 7-aaD and analyzed on a BD C6 Accuri flow cytometer. Early apoptotic (AnnexinV-PE positive, 7-aaD negative) events are represented graphically using Graph Pad Prism 6. In short, cells were resuspended in phosphate-buffered saline (PBS) containing 4 mM CaCl_2_, annexin V– phycoerythrin (PE; BD Pharmingen, San Jose, CA), and 7AAD according to the manufacturer protocol. Cells were analyzed by flow cytometry (Accuri C6)^10^.

### Mitochondrial Depolarization Assay

Mitochondrial Potential was measured using 200 nM TMRE (tetramethylrhodamine, ethyl ester) staining performed in triplicate and a representative image is shown. As a control for depolarization, cells were briefly treated with 20 µM FCCP (carbonyl cyanide 4-(trifluoromethoxy) phenylhydrazone) before adding TMRE. (Abcam, Cambridge, United Kingdom) Cells were collected on a BD Accuri C6 flow cytometer.

### Biochemical Analysis

We tested the effects of paclitaxel alone or in combination with BKM120 on our panel of two ovarian cancer cell lines. We performed immunoblots by Western blots on the equivalent amounts of protein (Bradford assay) using clarified cell lysates as mentioned earlier^11^. Normalized lysates (20–40 μg protein) were resolved by 10% sodium dodecyl sulfate-polyacrylamide gel electrophoresis for Western blot^10^. Doses of BKM120 (500 nM and 1 µM) and paclitaxel (2.5 nM and 5 nM) were used for the validation study. Uncropped blots (molecular weight markers labeled on the blot) are available in the Supplementary Figure. We have determined the semi-quantitative WB expression of the proteins by Image-J^10^.

### Live/Dead Cell Assay

For the validation purpose, we performed a fluorescence-based assay of Slive/dead cells using fluorescence kit from Invitrogen (Molecular Probes, Grand Island, NY) according to the manufacturer’s protocol. In short, treated cells grown in 24-well plates (non-treated and treated) were washed with PBS and incubated with a 0.5 μM Eth-D-1 (Ethidium Homodimer-1) and 0.5 μM Calcein AM solution in PBS for 30 minutes at room temperature. Pictures were taken using an Olympus DP72 digital camera^10^.

## Results

### Mechanism-based triple fluorescence in live cancer cells following paclitaxel and BKM120

**Figure 1:**
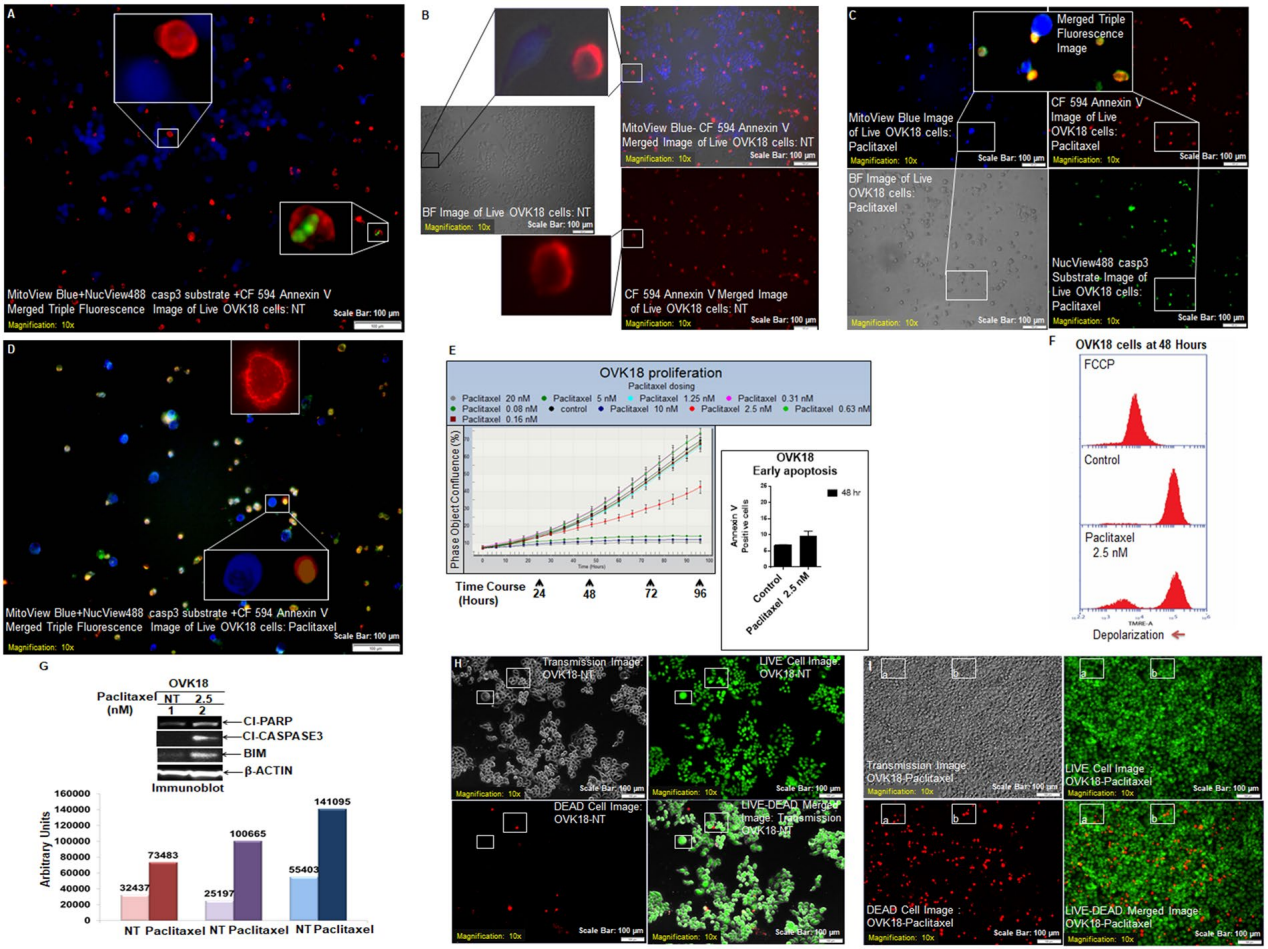
Live cell triple-fluorescence in paclitaxel-treated OVK18. OVK18 cells, control and treated were stained with MitoView Blue + NucView488 casp3 substrate +CF 594 Annexin V (**A**–**D**). Photomicrographs were taken from live cells in culture within a span of 10 minutes. In separate experiments, the validation of the effect of paclitaxel (2.5 nM) in OVK18 cells was tested by real-time proliferation (**E**, left panel), apoptosis (**E**, right panel), mitochondrial potential (**F**), expression of apoptotic markers by WB (**G**) and staining of live-dead cells (**H**,**I**). The expression of apoptotic markers by WB was semi-quantified using Image-J and presented in a bar diagram with arbitrary units. A representative of uncropped blot representing the expression of proteins (cleaved PARP1, cleaved caspase3, BIM, and beta-actin) by WB from an independent experiment is presented in the Supplementary Figure.

To evaluate mechanism-based apoptosis using triple fluorescence in live cancer cells we treated two ovarian cancer cell lines, OVK18 and A2780 with paclitaxel alone or in combination with BKM120 for 24 hours and tested the fluorescence localization of three critical component of live and apoptotic cells. Using MitoView Blue, we stained active mitochondria while using NucView488 casp3 we stained active caspase3 and CF 594 AnnexinV was used to localize plasma membrane asymmetry, flipped phosphatidylserine, a characteristic feature of apoptotic cells. A merged triple fluorescence image of live and non-treated (NT) OVK18 cells showed functional mitochondrial stained with bright blue (B). Fewer cells showed membrane fluorescence in red for CF 594 AnnexinV (R) and even fewer cells with green fluorescence for NucView488 casp3 (G) which stained active caspase3 (Fig. 1A). We observed that blue cells are mutually exclusive to both R and G cells when compared to the bright field image (BF) (upper and middle insets of Fig. 1B). R and G cells are sometimes co-localized (Inset of Fig. 1A) and sometimes not co-localized (lower inset of Fig. 1B). In paclitaxel-treated wells, the number of B cells is comparatively lesser in number than R and G cells (Fig. 1C). However, the property of mutual exclusivity of B cell to R and G cells is preserved as shown in the merged images (Fig. 1C,D). Figure 1D also shows a characteristic annexin V stain (inset) observed in paclitaxel-treated cells.

**Figure 2:**
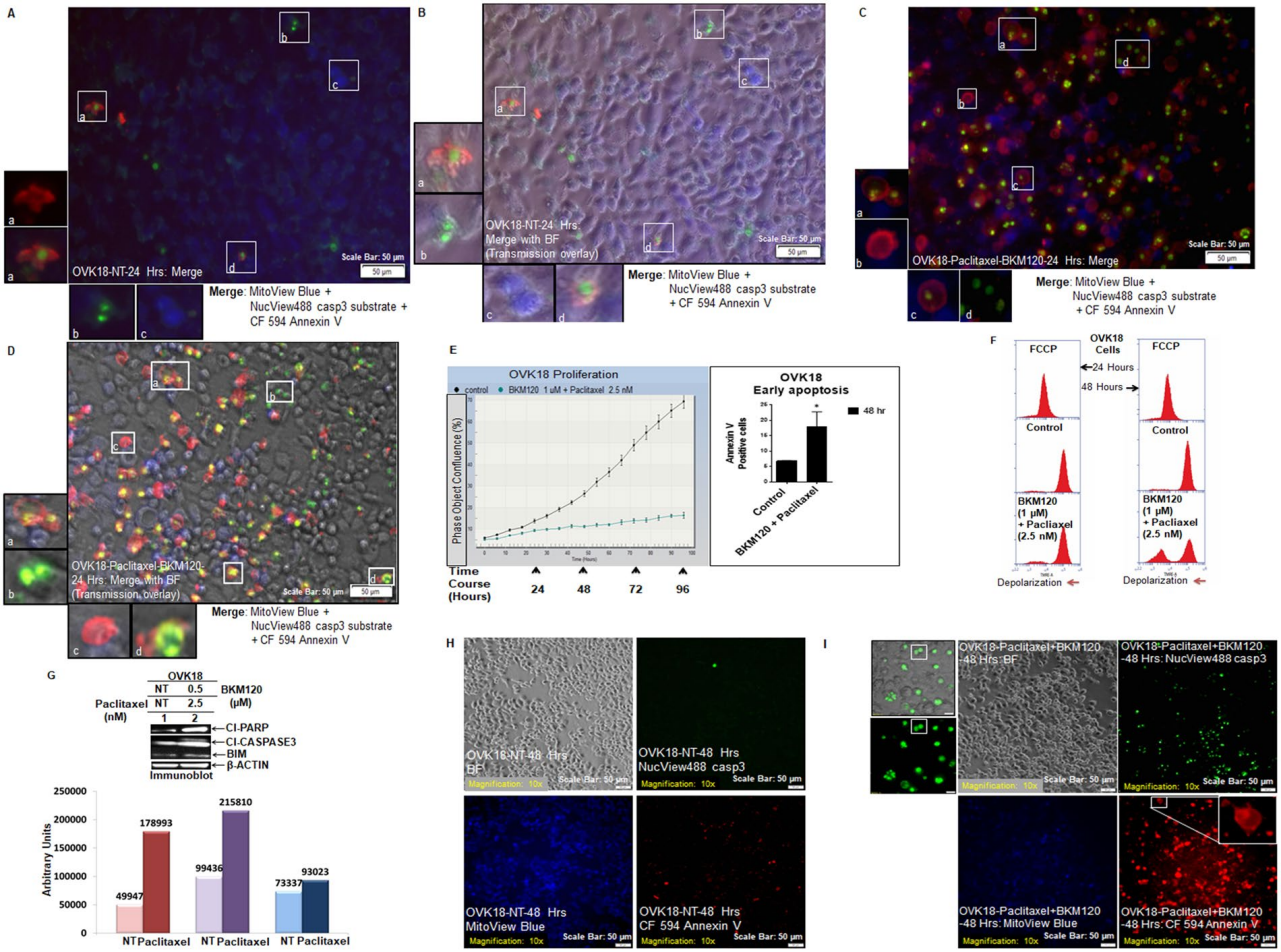
Live Triple-fluorescence in OVK18 cells treated with paclitaxel plus BKM120. Live OVK18 cells, control and treated were stained with MitoView Blue + NucView488 casp3 substrate +CF 594 Annexin V (**A**–**D**). Photomicrographs were taken from live cells in culture within a span of 10 minutes. In separate experiments, the validation of the effect of paclitaxel (2.5 nM) plus BKM120 (1 µM) was tested by real-time proliferation (**E**, left panel), apoptosis (**E**, right panel), the mitochondrial potential (**F**), and expression of apoptotic markers by WB (**G**). The expression of apoptotic markers by WB was semi-quantified using Image-J and presented in a bar diagram with arbitrary units. The cleaved caspase3 activity, annexin V positivity and mitochondrial status of non-treated (**H**) and treated (**I**) live OKK18 cells at 48 hours are presented. A representative of uncropped blot representing the expression of proteins (cleaved PARP1, cleaved caspase3, BIM, and beta-actin) by WB from an independent experiment is presented in the Supplementary Figure.

In separate experiments, we treated the OVK18 cells with paclitaxel and BKM120. The doses of paclitaxel and BKM120 were chosen from our previous reports (*Genomic landscape of the PI3K-AKT pathway and the RAS-RAF-MEK-ERK pathway in ovarian cancer: A treatment strategy*, *Pradip De*, *et al*., *WIN 2016 June 27–28*, *Paris*, *France*, *Abstract # P4*.*3*; *Genomic landscape of the PI3K pathway and cell-cycle pathway in ER*+ *BC: A treatment strategy Pradip De*, *et al*., *14th*. *St*. *Gallen International Breast Cancer Conference*, *Vienna*, *Austria March 18–21st*. *2015 Abstract #404; Molecular aberrations of the PI3K-AKT-mTORC1/C2 pathway in ovarian cancers: A strategy for targeted therapy*, *Pradip De*, *et al*., *AACR-NCI-EORTC Molecular Targets and Cancer Therapeutics Conference 2017*, *Philadelphia*, *PA*, *Abstract #462*). The staining characteristics of NT OVK18 cells were observed to be comparable as shown in Fig. 2A,B. The selected cells (a, b, c, d) from the field in Fig. 2A,B are boxed in the photomicrograph and presented as insets of merged images with (Fig. 2B) or without (Fig. 2A) the transmission overlay to demonstrate the mutual exclusivity of B cells from R and/or G cells as well as occasional co-localization of R and G fluorescence in apoptotic cells which never show a bright blue signal from a functional mitochondria. Figure 2C,D showed higher numbers of R and G apoptotic cells in treated wells while the mutual exclusivity property of the staining is preserved. The selected cells (a, b, c, d) from the field in Fig. 2C,D are boxed in the photomicrograph and presented as insets of merged images with (Fig. 2D) or without (Fig. 2C) the transmission overlay to demonstrate the mutual exclusivity of B cells from R and/or G cells as well as frequent co-localization of R and G fluorescence in apoptotic cells.

**Figure 3:**
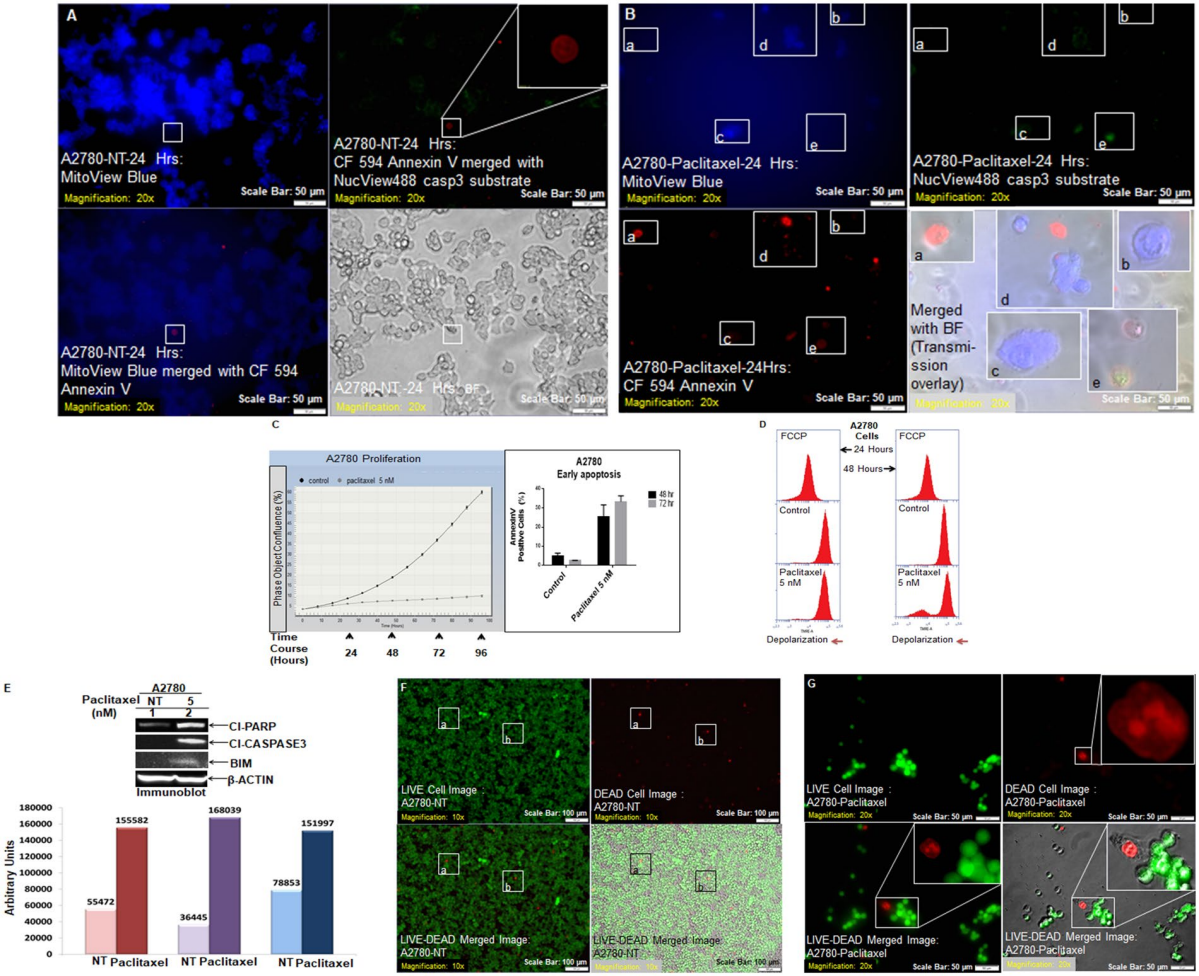
Live Triple-fluorescence in A2780 cells treated with paclitaxel. Live A2780 cells, control and treated were stained with MitoView Blue + NucView488 casp3 substrate +CF 594 Annexin V (**A**,**B**). Photomicrographs were taken from live cells in culture within a span of 10 minutes. In separate experiments, the validation of the effect of paclitaxel (5 nM) was tested by real-time proliferation (**C**, left panel), apoptosis (**C**, right panel), the mitochondrial potential (**D**), expression of apoptotic markers by WB (**E**) and staining of live-dead cells (**F**,**G**). A representative of uncropped blot representing the expression of proteins (cleaved PARP1, cleaved caspase3, BIM and beta-actin) by WB from an independent experiment is presented in the Supplementary Figure.

We also tested the triple fluorescence staining in A2780 ovarian cancer cell line with or without paclitaxel (Fig. 3). A2780 cells under NT condition had a high number of B cells indicating functional mitochondria and a few R cells which were mutually exclusive to B cells (Fig. 3A). While paclitaxel-treated A2780 cells were significantly devoid of B stains although the mutual exclusivity property of B cell to R and/or G cells are preserved as shown with the help of transmission overlay images (Fig. 3B, insets). A representative of uncropped blot representing the expression of proteins (cleaved PARP1, cleaved caspase3, BIM, and beta-actin) by WB from an independent experiment is presented in the Supplementary Figure.

### Validation of Mechanism-based triple fluorescence in live ovarian cancer cells following paclitaxel and BKM120

To establish our staining, we performed extensive validation protocol to prove the effect of paclitaxel alone or in combination with BKM120 in OVK18 and A2780 cells using (1) a quantitative confluence, (2) AnnexinV-PE staining (3) mitochondrial potential, (4) expression of apoptotic signals (cl-caspase3, Cl-PARP and BIM protein) and (5) fluorescent imaging of cells using simultaneous live/dead markers (Calcein AM green/EthD-1 red). The above set of extensive validation criteria was used for all three sets of experiments which tested different cell lines as well as different drug treatments as shown in Fig. 1 (paclitaxel treatment in OVK18 cells), Fig. 2 (paclitaxel plus BKM120 in OVK18 cells) and Fig. 3 (paclitaxel treatment in A2780 parental cells). In the Fig. 1 we showed that real-time proliferation was dose-dependently blocked in OVK18 cells following treatment with paclitaxel (E, left panel) which led to the apoptosis at 48 hours (E, right panel) and loss of mitochondrial potential (F), with simultaneous expression of apoptotic markers, cleaved caspase3, cleaved PARP and BIM by WB (G). Finally, we showed increased in dead cells staining following paclitaxel as compared to NT condition (H–I).

In the Fig. 2 we showed that real-time proliferation was blocked in cells following paclitaxel plus BKM120 treatment (E, left panel) which led to the apoptosis at 48 hours (E, right panel) and loss of mitochondrial potential at 24 and 48 hours (F), with simultaneous expression of apoptotic markers, cleaved caspase3, cleaved PARP and BIM by WB (G). Since we presented our protein expression, flow cytometry, and real-time proliferation data at 48 hours and 72 hours, we also tested cleaved caspase3 activity, annexin V positivity and mitochondrial status at 48 hours under similar treatment conditions. The cleaved caspase3 activity, annexin V positivity and mitochondrial status were also examined at 48 hours following the treatment of live OVK18 cells with paclitaxel and BKM120 (Fig. 2H,I).

In the Fig. 3 we showed that real-time proliferation was blocked in parental A2780 cells following paclitaxel (C, left panel) which led to the apoptosis at 48 and 72 hours (C, right panel) and loss of mitochondrial potential at 24 and 48 hours (D), with simultaneous expression of apoptotic markers, cleaved caspase3, cleaved PARP and BIM by WB (E). Finally, we observed an increase in the number of dead cells the following paclitaxel as compared to NT condition (F–G).

### Co-Localization study based on the characteristic pattern of co-staining of live tumor cells with the fluorescent markers NucView488 Casp3 substrate, CF594 AnnexinV, and MitoView Blue

**Figure 4:**
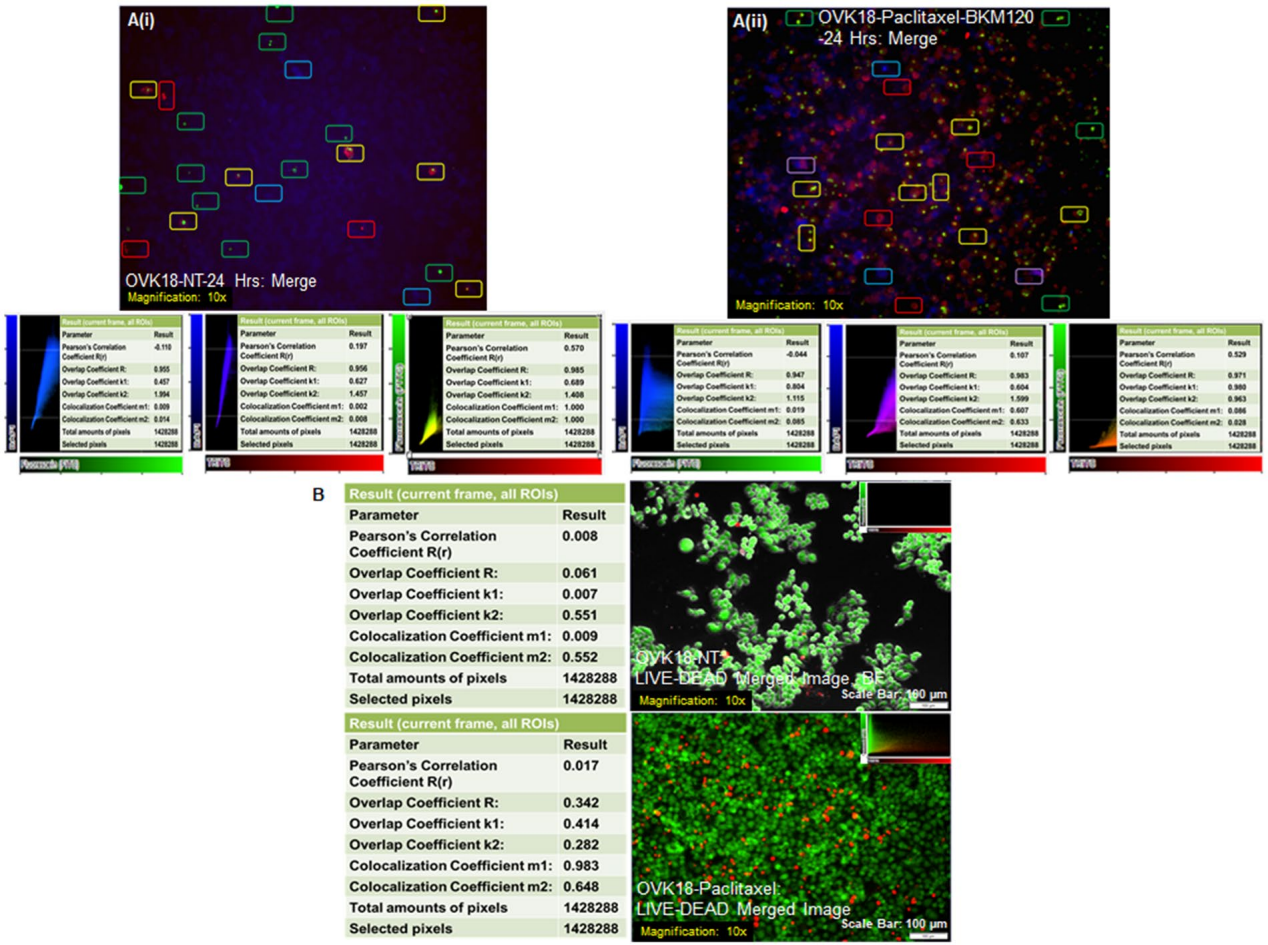
Pixel co-localization between FITC (NucView488 casp3 Substrate and Calcein AM), DAPI (MitoView Blue) and TRITC (CF 594 Annexin V and EthD-1) channels from live cell images of paclitaxel (2.5 nM) + BKM120 (500 nM) treated OVK18 cells stained for triple fluorescence (**A**) and paclitaxel (2.5 nM) treated live-dead OVK18 cells (**B**) was presented. (**A**) Co-localization between FITC, DAPI, and TRITC channels was determined in the scatterplot (inset) of merged (channels). Image applied to all frames with a target area of whole frames (total 1428288 pixels and selected1428288 pixels). Pixel co-localization between DAPI–FITC (upper inset), DAPI–TRITC (middle inset), and FITC – TRITC (lower inset), channels with co-efficient parameters are presented (insets) in non-treated (NT; Ai) and drug-treated (Paclitaxel-BKM120; Aii) OVK18 cells. A few cells with red and green stain are marked with yellow boxes, and cells with red and blue stain are marked with violet boxes. A few cells with red, blue, and green stains are marked with red, blue, and green boxes respectively. The co-location pattern of three channels are found to be characteristically different between NT and treated group. (**B**) Co-localization between FITC and TRITC channels was determined in the scatterplot (inset) of merged (channels). Image applied to all frames with a target area of whole frames (total 1428288 pixels and selected1428288 pixels). Upper panel represents non-treated (NT) live-dead OVK18 cells. Pearson’s Correlation Coefficient R(r) [Whole Frame] was 0.017 (A perfect positive linear relationship, r = 1). Overlap Coefficient was 0.342. Overlap Coefficient K1 [Whole Frame] was 0.414 and K2 [Whole Frame] 0.282. Co-localization co-efficient M1(whole frame) was 0.983 M2 (whole frame) was 0.648. The lower panel represents paclitaxel-treated (Paclitaxel) live-dead OVK18 cells. Co-localization between FITC and TRITC channels was determined in the scatterplot (inset) of merged (channels). Image applied to all frames with a target area of whole frames (total 1428288 pixels and selected1428288 pixels). Pearson’s Correlation Coefficient R(r) [Whole Frame] was 0.008. (A perfect positive linear relationship, r = 1). Overlap Coefficient was 0.061. Overlap Coefficient K1 [Whole Frame] was 0.007, and K2 [Whole Frame] was 551. Co-localization co-efficient M1(whole frame) was 0.009 M2 (whole frame) was 0.552.

We determined the pixel co-localization between FITC (NucView488 casp3 Substrate and Calcein AM), DAPI (MitoView Blue) and TRITC (CF 594 Annexin V and EthD-1) channels from live cell images of paclitaxel (2.5 nM) + BKM120 (500 nM) treated OVK18 cells stained for triple fluorescence (Fig. 4A) and paclitaxel (2.5 nM) treated live-dead OVK18 cells (Fig. 4B) using OLYMPUS cellsSens Dimension Desktop Version 1.18 imaging software.

Co-localization between FITC, DAPI, and TRITC channels was determined in the scatterplot (inset) of merged (channels). The image was applied to all frames with a target area of whole frames (total 1428288 pixels and selected1428288 pixels). Channels with co-efficient parameters are presented (insets) for non-treated (NT; Fig. 4Ai) and drug-treated (Paclitaxel-BKM120; Fig. 4Aii) OVK18 cells. Pixel co-localization between DAPI – FITC (Fig. 4Ai, upper inset), DAPI – TRITC (Fig. 4Ai, middle inset), and FITC – TRITC (Fig. 4Ai, lower inset) of NT cells show a minimum overlap between B cells and R and/or G cells. A few cells with red and green stain are marked with yellow boxes, and cells with red and blue stain are marked with violet boxes. A few cells with red, blue, and green stains are marked with red, blue, and green boxes respectively. The co-location pattern of three channels are found to be characteristically different between NT and treated group. Results show that when R or G channel was plotted against B channel the Pearson’s CorrelationCoeffient R(r) indicated a minimum co-localization as shown in Fig. 4Ai (upper and middle insets). In contrast, when R channel was plotted against G channel, a reasonable co-localization was observed (co-localization co-efficient m1 and m2 values are 1) as shown in the lower inset of Fig. 4Ai). A similar trend is observed for the Pearson’s Correlation Coefficient R(r) in treated cells as shown in Fig. 4Aii (upper, middle and lower insets). While cells with both R and G stain was observed in both NT and treated cells, the B cells were found to be mutually exclusive to R and/or G cells in the NT cells. Interestingly, however, we observed cells with red and blue stains (are marked with violet boxes) characteristically in only treated cells.

We also determined co-localization between FITC and TRITC channels in the scatterplot (inset) of merged (channels) for the LIVE-DEAD cells in paclitaxel-treated OVK18 cells (Fig. 4B). Upper panel (Fig. 4B) represents non-treated (NT) live-dead OVK18 cells. Pearson’s Correlation Coefficient R(r) [Whole Frame] was 0.017 (A perfect positive linear relationship, r = 1). Overlap Coefficient was 0.342. Overlap Coefficient K1 [Whole Frame] was 0.414 and K2 [Whole Frame] 0.282. Co-localization co-efficient M1(whole frame) was 0.983 M2 (whole frame) was 0.648. Lower panel (Fig. 4B) represents paclitaxel-treated (Paclitaxel) live-dead OVK18 cells. Co-localization between FITC and TRITC channels was determined in the scatterplot (inset) of merged (channels). Image applied to all frames with a target area of whole frames (total 1428288 pixels and selected1428288 pixels). Pearson’s Correlation Coefficient R(r) [Whole Frame] was 0.008. (A perfect positive linear relationship, r = 1). Overlap Coefficient was 0.061 Overlap Coefficient K1 [Whole Frame] was 0.007, and K2 [Whole Frame] was 551. Co-localization co-efficient M1(whole frame) was 0.009 M2 (whole frame) was 0.552. Data showed that no co-localization between R (Red cells presents dead cells stained with EthD-1 in separate experiments) and G (Green cells presents live cells stained with Calcein AM in separate experiments).

**Figure 5:**
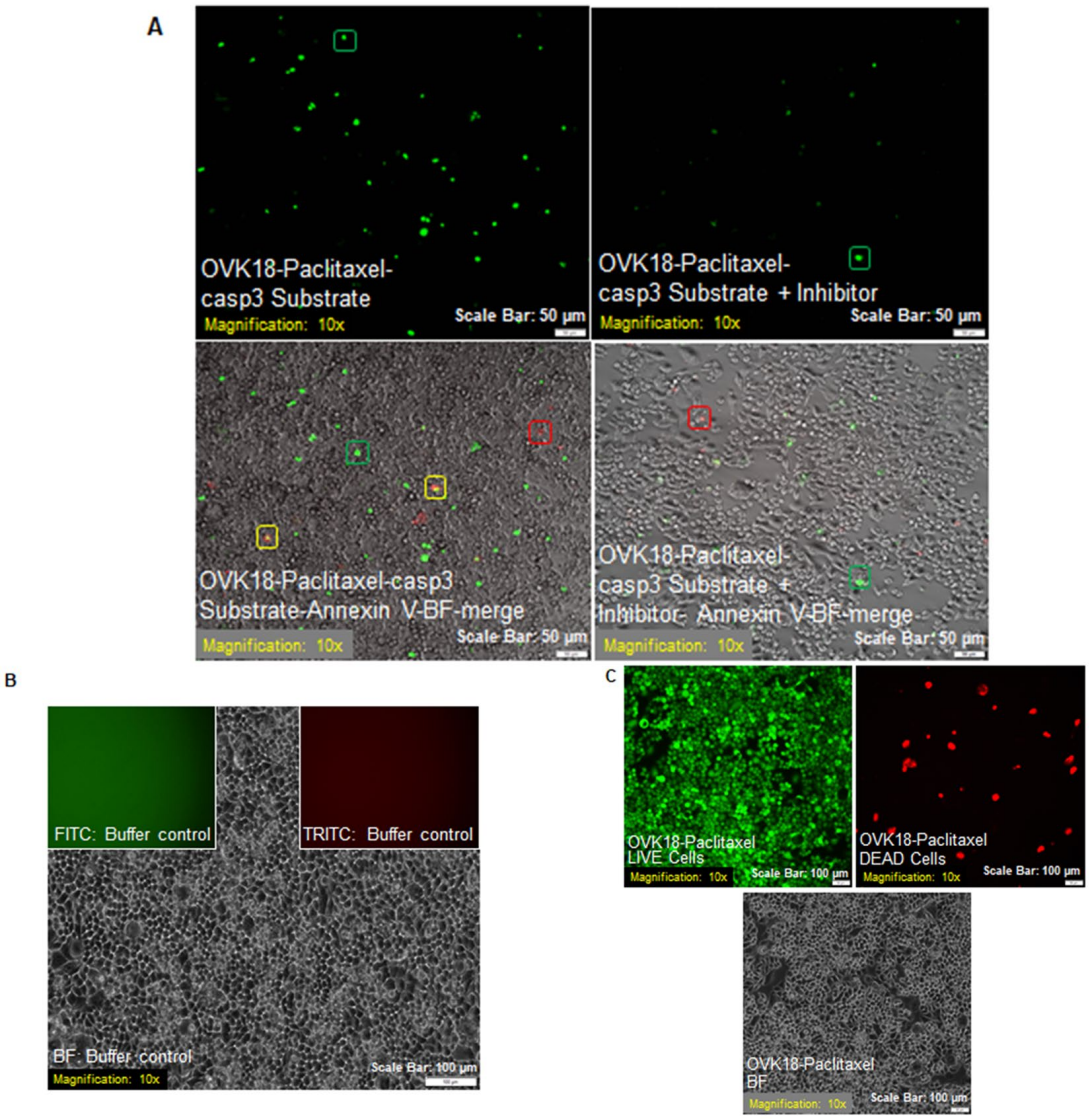
Effect of a competitive inhibitor for caspase-3/7, Ac-DEVD-CHO on the caspase-3/7 activity in live apoptotic cells following paclitaxel treatment. Cells were first treated with paclitaxel for 24 hours and then stained for NucView488 and CF 594 Annexin V in the presence of Ac-DEVD-CHO (**A**). Live cells treated with Ac-DEVD-CHO specifically inhibited caspase-3/7 activity. A buffer control and live-dead image of cells following the treatment with paclitaxel were shown in (**B**,**C**) respectively.

The Ac-DEVD-CHO, a synthetic tetrapeptide (containing the amino acid sequence of the PARP cleavage site) that acts as a competitive inhibitor for caspase-3/7 was used to inhibit caspase-3/7 activity in apoptotic cells, and to study events downstream of caspase-3/7 activation (Fig. 5). Figure 5A showed OVK18 cells which were previously exposed to paclitaxel for 24 hours when treated with Ac-DEVD-CHO exhibited significant inhibition of nuclear staining (green; as presented in the upper right panel of the figure). In contrast, the absence of the inhibitor caused an appreciable increase in the cleaved caspase3 activity (green cells as presented in the upper left panel of the figure) when tested on paclitaxel-treated OVK18 cells. The lower panel (right) of the figure demonstrated that the presence of the inhibitor also perturbed the annexin V staining, a downstream event of caspase3 activation in OVK18 cells as compared to the cells which were not exposed to the inhibitor (lower left panel). A buffer control and live-dead image of cells following the treatment with paclitaxel were shown in Fig. 5B,C respectively.

## Discussion

Morphological and biochemical characteristics of apoptosis form the basis of identification of apoptosis in cells. Based on methodology, apoptosis assays are classified into six major groups, cytomorphological alterations, DNA fragmentation, detection of caspases, cleaved substrates, regulators and inhibitors, membrane alterations, detection of apoptosis in whole mounts and mitochondrial assays^2^.

Our study provides a research tool to morphologically connect critical events of apoptosis, its cellular machinery, with its signaling pathways in tumor cells following chemotherapy and targeted drug. Triple-fluorescence staining in live tumor cells brings out the mechanistic relationship between different rate-limiting biochemical features of apoptosis (like the mitochondrial potential, activation/enzymatic function of executioner caspase, caspase3) and what these features functionally links to namely morphological features (like the loss of lipid asymmetry and phosphatidylserine exposure to the extracellular space). We have used (1) real-time quantitative measure of confluency (Incucyte), (2) quantification of apoptosis by Annexin V (Flow Cytometry), (3) cellular expression of apoptosis signaling markers like cl-caspase3, cl-PARP, and BIM, (Western blot), (4) live-dead cell assay and (5) mitochondrial potential (TMRE-A based measure of mitochondrial depolarization) for the validation of the staining.

The choice of our triple fluorescence stainings was based on three cardinal and sequential features of apoptosis/live cell which are mutually exclusive. *First*, we chose to stain for mitochondrial function (a feature of live cell/non-apoptotic cell) and validated the staining by measuring TMRE-based mitochondrial membrane potential under same treatment condition in the same cell lines (Figs 1F, 2F and 3D). The more negative the ΔΨm more is the accumulation of TMRE, a cationic red-orange fluorescent dye in the active mitochondria indicating the liveliness of cells. Depolarized or inactive mitochondria have decreased membrane potential and fail to sequester TMRE so that the apoptotic cells exhibit weak orange fluorescence.

*Second*, we chose to stain for the enzyme activity of executioner/effector caspase3 (a biochemical event of apoptotic cells merging both intrinsic and extrinsic pathways) in live cells and validated the staining by determining the semi-quantitative WB expression of (1) cleaved caspase3 (active form of the enzyme), (2) cleaved PARP (PARP is a physiological substrate of caspase3, caspase-3 is primarily responsible for the cleavage of PARP during cell death) and (3) BIM (Pro-apoptotic Bcl-2 family member and is degraded post-translationally through ubiquitin/proteasome pathways in a caspase-3-dependent manner)^12,13^ (Figs 1G, 2G and 3E). We used caspase3 substrate for real-time apoptosis detection. Once the apoptotic program reached its penultimate stage, the executioner caspases, caspases-3 and -7 were able to cleave and activate Xrp8 protein, which acts as an apoptosis-induced lipid scramblase and leads to the loss of lipid asymmetry, resulting in phosphatidylserine exposure to the extracellular space^14^ and activation of ROCK I by caspase-3 has been reported to play a role in bleb formation in apoptotic cells^15^. Hence we have incorporated fluorescence detection for the enzyme activity for caspase3 with the fluorescence detection of phosphatidylserine moiety on the outer leaflet of apoptotic cells in live culture conditions. To test the specificity of the cl-caspase3 enzymatic activity, we used Ac-DEVD-CHO, a competitive inhibitor for caspase-3/7 (Fig. 5) on paclitaxel-treated live OVK18 cells. As expected the inhibitor blocked the activity of the enzyme and its downstream effector, Annexin V.

*Third*, we chose to stain for the membrane asymmetry due to flipped phosphatidylserine (an “eat me” signal for the scavenging macrophages) and validated the staining by determining the flow cytometry-based detection of annexin V in cells (right panel of Figs 1E, 2E and 3C) and its inverse relationship with cell proliferation in real-time (left panel of Figs 1E, 2E and 3C). Phagocytic uptake of apoptotic cells is the last event of apoptosis and caspase-3 is one of the caspases reported to be involved in the regulation of phosphatidylserine externalization during oxidatively stressed erythrocytes^2^. The asymmetrical composition of the outer and inner leaflets of the plasma membrane comprised of phosphatidylcholine and sphingomyelin on the outer leaflet while phosphatidylserine, phosphatidylinositol, and phosphatidylethanolamine on the inner leaflet. Lipid asymmetry defines the curvature and electrochemical properties of the plasma membrane as well as provides a correct locational presentation of the determined lipids of a particular type of cell signal, a scavenging signal in this case. The exposure cell surface phosphatidylserine is a classic “eat me” feature that signals phagocytosis of postapoptotic bodies. The flipped appearance of phosphatidylserine moiety on the outer leaflet of apoptotic cells facilitates noninflammatory phagocytic recognition for their early uptake and disposal^16^. Annexin V is a recombinant phosphatidylserine-binding protein which interacts strongly and specifically with phosphatidylserine residues and classically used for the detection of apoptosis^17^.

It should be considered that not all of the biochemical events mentioned here for apoptosis are specific to apoptosis only, and events that occur in apoptotic tumor cells are a stage-dependent, time-dependent and stimuli/drug-dependent in a particular context of gene expression. The strength of the staining protocol described in this study is its easy one-step nature to stain three critical morphological and biochemical feature/events of apoptotic cells simultaneously in contrast to a non-apoptotic cell in a mutually exclusive manner as determined by the colocalization coefficients (Fig. 4). The limitation of the protocol is that it is not necessarily quantitative but mechanistic. The staining protocol presented here does not provide any form of quantification. The inability of stains to quantify apoptosis is related to the inherent nature of the live cell staining and the property of the apoptotic cells to get loosened from the plate as the cell reaches the final stage of the apoptosis. The purpose here was to provide easy and informative staining identifying critical features of alive versus apoptotically dead cells following the treatment of anti-cancer drugs. However, the stain can be applied to any relevant drug treatment as long as the user is aware of the strength and limitation of the staining. We have used this staining in breast cancer cell lines with different drug combinations in our laboratory (data not shown).

Loss of apoptotic signaling is a hallmark of cancer. The mechanism of apoptosis induction has been a feature utilized to characterize and classify anti-tumor drugs including chemotherapeutic agents and pathway-targeted drugs in determining *ex vivo* and *in vitro* efficacy. The ability to regulate apoptosis in tumor cells is recognized for its immense therapeutic potential in oncology. Induction of apoptosis in tumor cells is one of the critical modes of action by which anti-tumor drugs including chemotherapy drugs or targeted therapy drugs work. Thus tools to study the mechanism of induction of apoptosis in tumor cells following drugs are important in identifying the mode of action of apoptosis-inducing anti-tumor agents in cancer research. Suppression of apoptosis during carcinogenesis play a central role in the development and progression of some cancers had been long reported by the same group of scientists who first identified apoptosis as a basic biological phenomenon with its implications in tissue kinetics^8^.

There are several well-established methods for the quantification of apoptosis. These published methods to quantify apoptosis on the basis of individual feature(s) of apoptosis, like annexin V or mitochondrial potential or active state of different caspases. However, all these methods quantify apoptosis on the basis of any one of the many features of apoptosis at one time (either annexin V or mitochondrial potential or active state of different caspases). Also, none of these methods can identify apoptosis in live cells. Our method identifies three cardinal features of apoptosis simultaneously in one live cell following drug treatment in a real-time manner. Our method establishes a staining protocol to study (1) enzymatic activation of the executioner caspase3/7, (2) its downstream signaling event of the exposure of phosphatidylserine on the cell surface in an apoptotic cell simultaneously with (3) the state of mitochondrial potential in the same cell. The strength of our study lies in the fact that we have been successful in coupling all three above mentioned features of apoptosis simultaneously in live cells in real time. To the best our knowledge this is the most stringent method to identify apoptosis conclusively in live cells. The staining protocol presented here will help in deciphering the mechanistic involvement of apoptosis following treatment with anti-cancer drugs in real time.

## Electronic supplementary material

Supplementary Figure
