# Supplementary material for: Triple Fluorescence staining to Evaluate Mechanism-based Apoptosis following Chemotherapeutic and Targeted Anti-cancer Drugs in Live Tumor Cells
Source: Sci Rep. 2018 Sep 4;8:13192. doi: 10.1038/s41598-018-31575-3 (PMC6123436; doi:10.1038/s41598-018-31575-3)
Supplement: Supplementary file 1 — Supplementary Figure [file 41598_2018_31575_MOESM1_ESM.pdf]

Triple Fluorescence staining to Evaluate Mechanism-based Apoptosis following Chemotherapeutic and Targeted Anti-cancer Drugs in Live Tumor Cells

Pradip De, Jennifer H. Carlson, Brian Leyland-Jones, Casey Williams, Nandini Dey

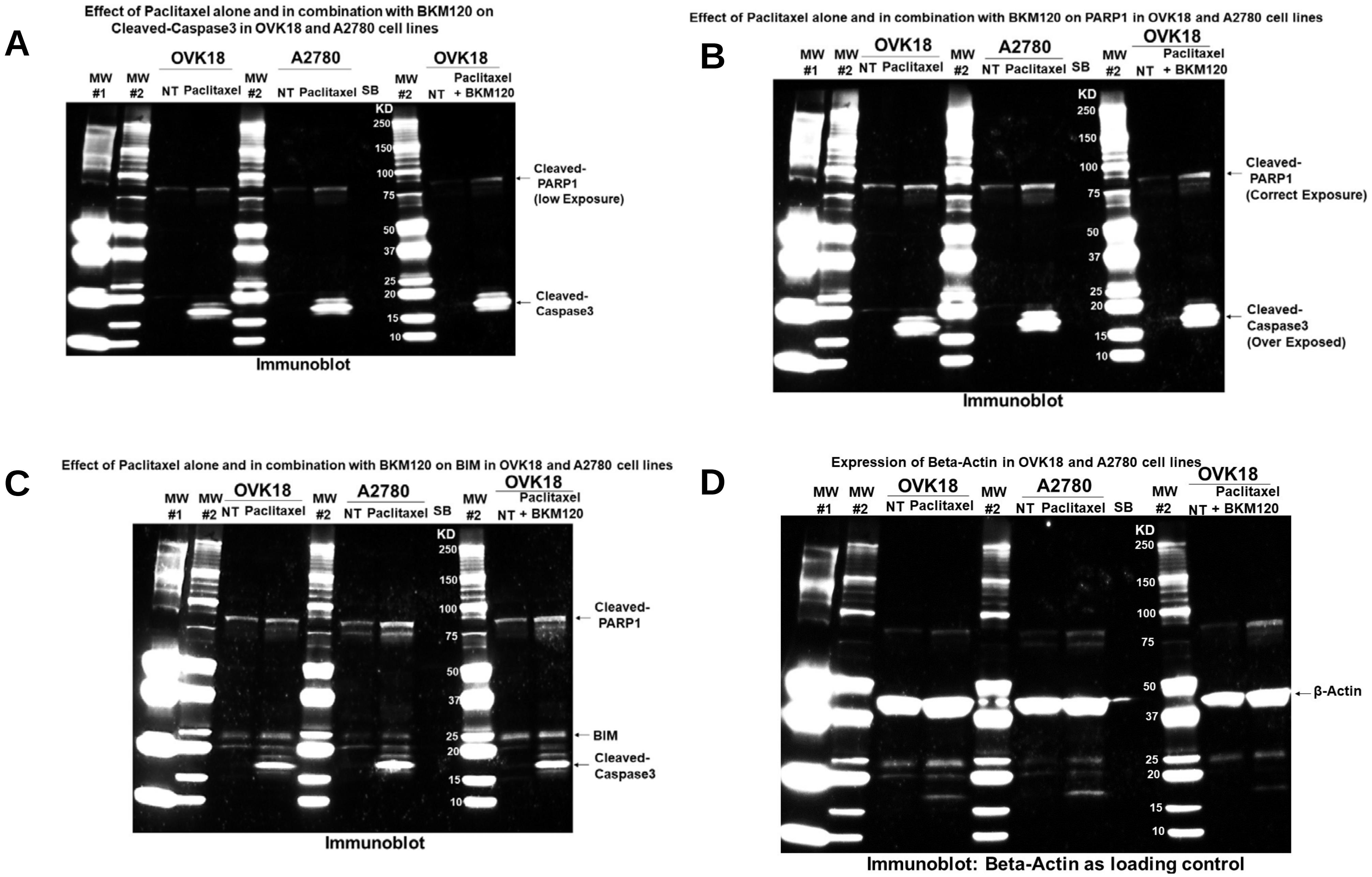

Supplementary Figure
